# Hollow plasmonic antennas for broadband SERS spectroscopy

**DOI:** 10.3762/bjnano.6.50

**Published:** 2015-02-18

**Authors:** Gabriele C Messina, Mario Malerba, Pierfrancesco Zilio, Ermanno Miele, Michele Dipalo, Lorenzo Ferrara, Francesco De Angelis

**Affiliations:** 1 Istituto Italiano di Tecnologia, Nanostructures Department, Via Morego 30, 16163 Genova, Italia

**Keywords:** biosensing, multiband field enhancement, plasmonics, Raman spectroscopy, SERS

## Abstract

The chemical environment of cells is an extremely complex and multifaceted system that includes many types of proteins, lipids, nucleic acids and various other components. With the final aim of studying these components in detail, we have developed multiband plasmonic antennas, which are suitable for highly sensitive surface enhanced Raman spectroscopy (SERS) and are activated by a wide range of excitation wavelengths. The three-dimensional hollow nanoantennas were produced on an optical resist by a secondary electron lithography approach, generated by fast ion-beam milling on the polymer and then covered with silver in order to obtain plasmonic functionalities. The optical properties of these structures have been studied through finite element analysis simulations that demonstrated the presence of broadband absorption and multiband enhancement due to the unusual geometry of the antennas. The enhancement was confirmed by SERS measurements, which showed a large enhancement of the vibrational features both in the case of resonant excitation and out-of-resonance excitation. Such characteristics indicate that these structures are potential candidates for plasmonic enhancers in multifunctional opto-electronic biosensors.

## Introduction

Cells are extremely complex systems that consist of hundreds of different molecules that can react and give rise to many different chemical processes. In addition to the complexity of the cellular chemical environment, it must also be considered that some of the biomolecules of interest are present in very low concentrations. Therefore, monitoring such an environment requires techniques that can offer both flexibility and high sensitivity for all cases. In other words, the investigation of the biological functions of living cells requires the acquisition of information from many different cell parts. For example, considering the most common investigation method in biology, namely fluorescence spectroscopy, it offers very high sensitivity, but at the cost of being sensitive only to those molecules for which the label has been applied to the bio-system. Most commonly, only a few selected components of the cells are stained and observed simultaneously by fluorescence microscopy: for example, membrane lipids together with the nucleus and cytoskeleton.

As an alternative to fluorescence methods, Raman spectroscopy is gaining attention as a technique for the study of live cells as it is a label-free non-invasive method and, moreover, it is not affected by photobleaching. Raman spectroscopy investigates the molecular bonds of all cell elements; therefore, it is potentially suitable for studying the entire cell environment without requiring the labelling of specific features of interest. However, the main drawback of Raman spectroscopy in this regard is the intrinsically low signal intensity, which leads to unsuitably high detection limits. In this respect, the exploitation of plasmonics for enhancing Raman signal has become an important factor for the routine application of this technique to biology.

There has been a significant increase in the number of studies concerning applications based on plasmonic effects in recent years. The areas of interest range from biomedicine, with examples of biosensing devices based on surface enhanced Raman scattering (SERS) enhancement [1–3], fluorescence [4–5], the surface plasmon resonance effect [6–7], mapping and imaging [8–10], to nanotechnology, with several works related to nanolithography [11–12], nanofocusing [13–14], nanolasers [15–16], waveguides [17–18] and magnetic field enhancement [19].

In these various disciplines, the rise of a trend targeting high performance spectroscopy techniques for biomolecules and cells can be recognized. Raman spectroscopy has already been implemented for whole live cell imaging [20] as well as its biological components [21].

It is well known that plasmons offer a great potential innovation in this regard because of their ability to capture and confine incident radiation in the so-called hotspots. To maximize this capability, plasmonic antennas can be designed to resonate at the desired excitation wavelength. As a consequence, they are efficient only in a limited spectral band around the tailored resonance wavelength, rather than being useful over the entire vis–NIR range, which is desirable for Raman spectroscopy of biological specimens.

On the other hand, the Raman spectroscopy fingerprint of cells includes over 1000 features [22] and many of them emit a much higher signal when excited at a specific wavelength. For this reason, there is the need for an enhancement method that allows for high enhancement values over a wide range of exciting wavelengths. With this, the same device can be exploited to perform highly sensitive SERS spectroscopy on all cell features using various excitation wavelengths. In a recent paper it was demonstrated that 3D hollow nanoantennas are capable of broadband absorption in the visible range of radiation [23].

In this work an experimental determination of the field enhancement supported by numerical calculations is presented. This shows how broadband absorption can be converted to multiband enhancement at the nanometer scale. This ability relies on the 3D nature of hollow vertically aligned (with respect to the metallic substrate plane) nanoantennas. The multiband behavior exhibited by these hollow nanoantennas may be a key factor for highly sensitive Raman measurements on the various components of the whole cell using an extended range of excitation light sources. Moreover, the 3D nature of these nanostructures allows them to be connected to electric power sources without disrupting the plasmon activity, thus opening their exploitation in the majority of the above cited fields.

## Results and Discussion

### Optimization of the electric field enhancement

**Figure 1:**
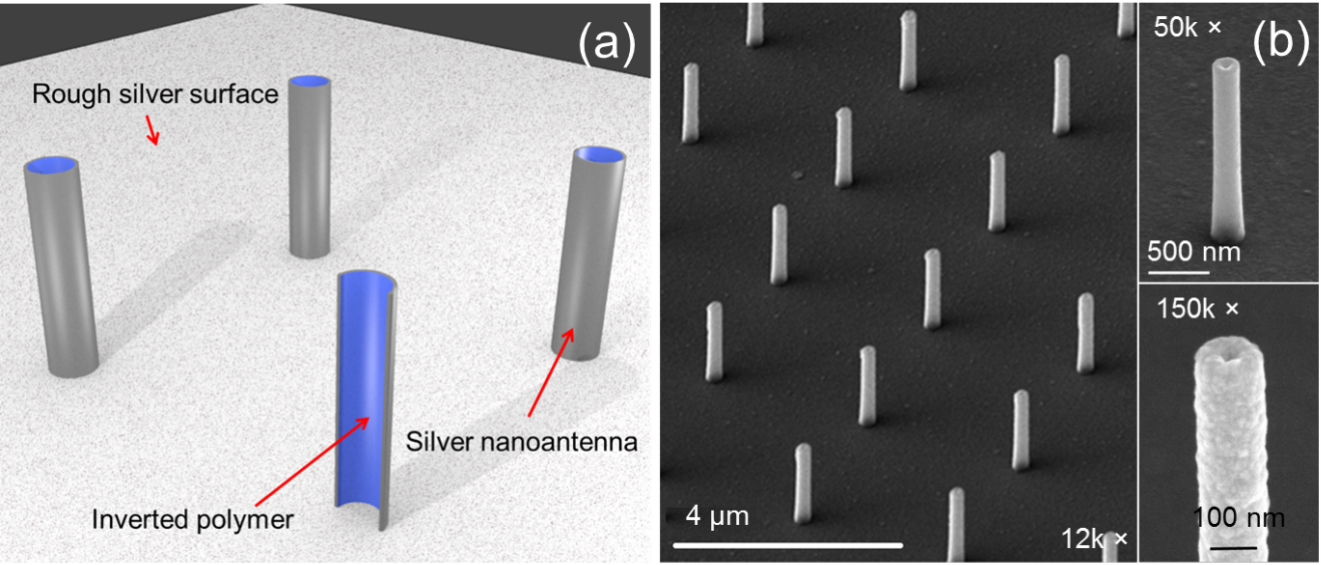
(a) Illustrative scheme representing the structure of a single antenna and (b) SEM images at different magnifications showing the array of antennas fabricated for this work.

The possibility of obtaining three-dimensional hollow nanoantennas with a high aspect ratio through an innovative technique based on secondary electron lithography was previously demonstrated [23]. Such a technique presents numerous advantages such as high versatility, allowing the production of structures with different sections, heights and diameters by controlling parameters such as the resist thickness and the beam energy. It is also conceivable to vary the angle of the ion milling, leading to the formation of tilted antennas. Moreover, the possibility of covering the polymer template with different materials expands the range of novel applications for such structures. In this paper, we exploit this fabrication approach to obtain cylindrical hollow antenna structures consisting of an inverted polymer covered with silver, as shown in Figure 1.

The inner cavity adds remarkable new functions (with respect to common cylindrical structures), namely: (i) efficient excitation of high order modes that results in an effective broadband absorption, (ii) the capability of concentrating the electric field in 3D hollow nanocavities, and (iii) the capability of connecting the plasmonic antennas to a metallic film (i.e., an electrode) without preventing their plasmonic function. In particular, the last point represents an important breakthrough, since it enables the combination of plasmonic with electrical functionalities: the antennas can be used also as nanoelectrodes for photovoltaic, electro-photochemical catalysis, optoelectronic and bio-electrochemical devices.

As a final remark, it should also be considered that the proposed method is fast with respect to other approaches regarding the fabrication of the plasmonic nanostructures [24–25] including those based on FIB milling. In fact, it has been calculated that fabrication on the order of 100k structures per hour with fine spatial control and geometrical accuracy [23] can be accomplished, suggesting the possibility of covering large areas such as chips, printed circuit boards (PCBs) or multielectrode array (MEA) surfaces. To fully understand the potential of nanofabricated arrays, numerical calculations based on finite element method analysis aimed at modeling the behavior of hollow antennas have been performed.

The goal of the simulations was to maximize the enhancement of the electric field in the visible range (centered at around λ = 630 nm) in terms of both field amplitude and bandwidth.

We performed scattering simulations of a single antenna illuminated by TM polarized light impinging at 5° with respect to normal. The simulation layout realistically reproduced the fabricated structure, as depicted in Figure 1a. The scattered field is absorbed by perfectly matched layers placed all around the structure (not shown). As a figure of merit, we considered the electric field in air sampled 1 nm above the upper edge of the antenna.

**Figure 2:**
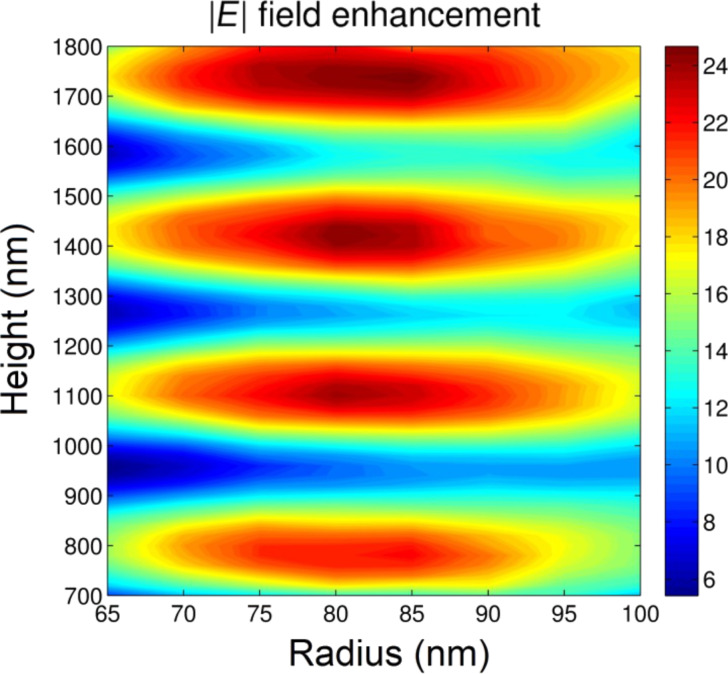
Electric field amplitude of silver antennas as a function of height and external radius. The maximum value of the field enhancement was found at an excitation wavelength of λ = 630 nm for *H* = 1400 and *R* = 80 nm.

In Figure 2, the values of field amplitude enhancement calculated for antennas whose height varies from 700 to 1800 and radius varies from 65 to 100 nm are shown. These parameter ranges have been chosen according to extended experiments and calculations (not reported for brevity). In order to save calculation time, we neglected the surface roughness in this optimization, whereas it will be considered later for the evaluation of the electric field enhancement. The model considers antennas with an 18 nm thick silver layer deposited on the surface. The field enhancement map shows several maxima as a function of the antenna height, which correspond to different orders of vertical Fabry–Perot resonances [23]. The calculations suggest that the maximum value of electromagnetic enhancement at around 630 nm excitation is 80 times the amplitude of the incoming electric field, and can be achieved for antennas of height *H* = 1400 nm and radius *R* = 80 nm.

Certainly, various geometries corresponding to different spectral bands of interest (both in terms of band position and width) are possible. Thus, by changing the device geometry, the optical response can be tuned according to specific needs. Therefore, the results presented here are considered to be representative of the general capabilities of this class of 3D devices. It is noted that the employment of antenna arrays could further tune and improve the optical response, especially in terms of absorption. However, here the properties of an isolated nanostructure are presented to better highlight its intrinsic features rather than those emerging from group properties.

**Figure 3:**
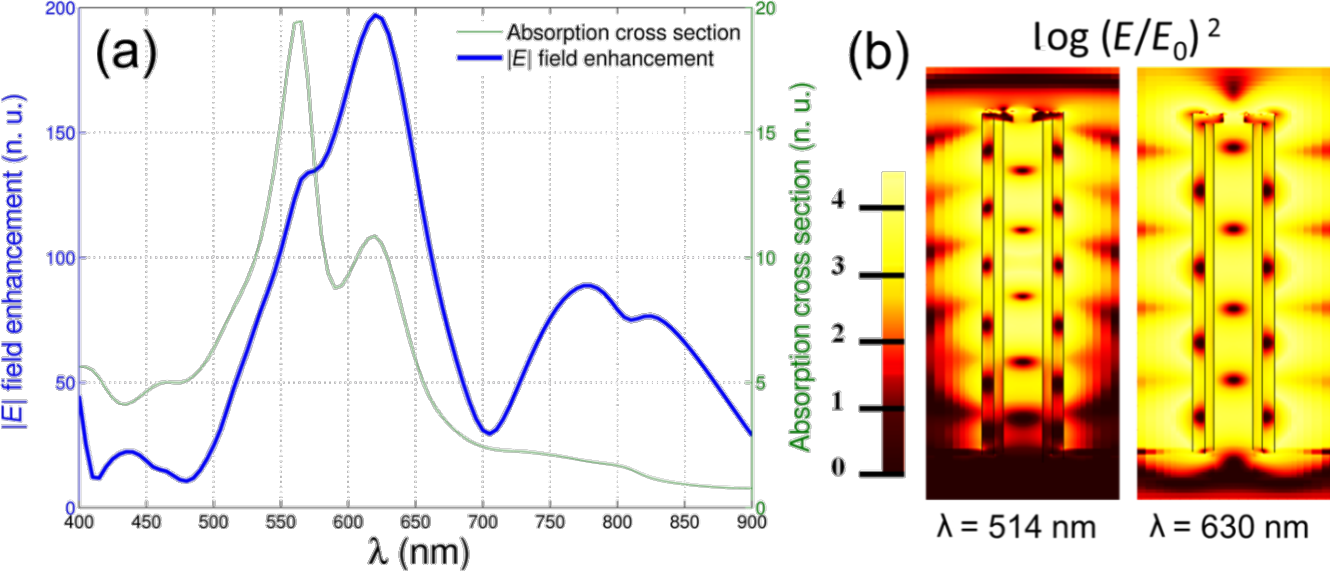
(a) Finite element method simulations of a silver nanotube with 1.4 µm height, 160 nm width, surface roughness of 4 nm and illuminated by TM polarized light impinging at 5°. The blue line represents the electric field enhancement calculated 1 nm above the upper antenna edge and normalized with respect to the impinging wave amplitude. The green line is the metal absorption cross section normalized to the incident power impinging on the antenna geometrical cross section. (b) Electric field maps corresponding to an excitation wavelength of 514 nm and 630 nm.

For the geometry optimized in this work (1.4 µm height and 160 nm width), the metal absorption and the enhancement in the visible range for a single antenna with a surface roughness of 4 nm on the tip was calculated. The results are reported in Figure 3 with the limited spectral range of 400–900 nm shown for clarity. The device exhibits very good performance regarding both the electric field enhancement and absorption.

Both spectra are characterized by numerous peaks, partially overlapped in a quasi-continuous profile. The electric field enhancement exhibits a minimum value of 50 times up to maximum of 200 times at 630 nm, as was expected from the optimization. However, the absorption only varies from a value of 20% in the NIR up to about 50% at 630 nm. Even though a complete analysis of the spectral features is not the main goal of this work, it can be noted that there is no one-to-one correspondence between absorption and field enhancement peaks. This behavior can be related to the fact that the electromagnetic radiation can penetrate inside the hollow nanocavity, as demonstrated in our previous work [23], thus generating absorption peaks that do not produce a field enhancement at the upper antenna edge. Indeed, the field enhancement inside the nanocavity has been estimated to be on the order of 20–25× the incident field [23].

It is worth noting that the obtained absorption and enhancement values are particularly high considering that they originate from a single antenna. This can be attributed to the complex three-dimensional geometry of the system. The efficient excitation of higher order modes leads to intriguing consequences from the point of view of absorption and enhancement. In fact, it should be considered that broadband resonances are commonly correlated to efficient excitation and absorption over the spectrum, but bring about a poor electric field enhancement, which depends on the plasmon lifetime. As a general rule, it is possible to assume Δω ≈ τ^−1^, where Δω is the resonance width and τ is the plasmon lifetime, hence a narrower resonance implies a more efficient electric field accumulation [26]. This is not possible in common planar antennas, which usually present only a few lower order resonances along the two main axes, namely the longitudinal and the transverse ones [27]. As a result, in accordance with our previous work [23], the hollow nanocylinder shows a continuous extinction profile where individual resonances are independently excited, yet may no longer be resolved. This behavior also results in a remarkable field enhancement over the whole visible range, as shown in Figure 3a.

For the sake of clarity, Figure 3b reports the electric field distributions (along the antenna cross-section) only at λ = 514 and λ = 630 nm. In both cases, the presence of hotspots localized over the antenna’s tip are clearly visible. These hotspots, together with the inner resonant modes (which give an additional contribution to the absorption), are correlated to the high electric field enhancement.

### Evaluation through SERS effect

According to the calculations reported in Figure 3, the presence of an electromagnetic hotspot at the tip of the antenna leads to the assumption that the plasmonic properties of the antennas will present a specific distribution along the nanotube height. To confirm such a hypothesis, measurements of Raman scattering intensities of a monolayer of cresyl violet dye have been performed while changing the focussing distance of the collection objective (100×, focus depth <500 nm) from the substrate, and thus the focus along the *z* direction (antenna main axis).

**Figure 4:**
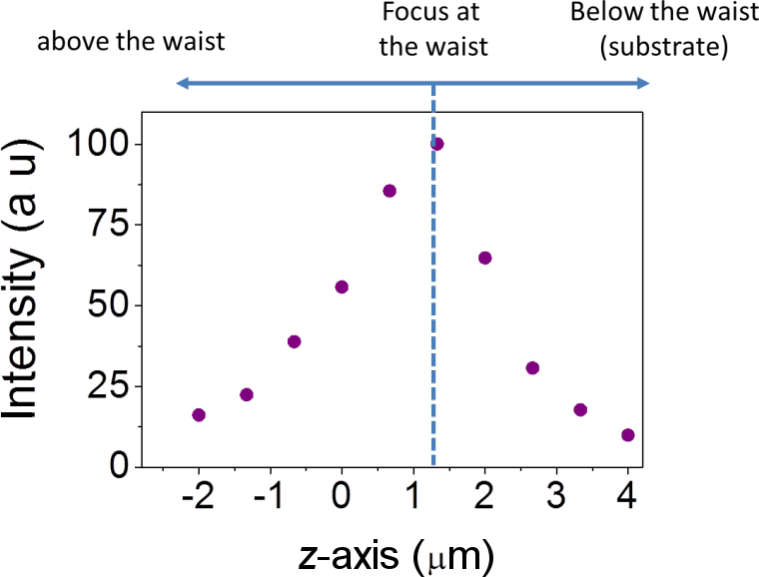
Measurements of Raman intensities along the *z*-axis of the antenna using cresyl violet dye.

To obtain a monolayer of dye, the sample was submerged into a solution of 1 µM cresyl violet for 5 min, allowing the dye molecules to be absorbed onto the metal surface. Then, the sample was rinsed in deionized water for 30 s to wash away excess molecules. The results of this experiment with an excitation wavelength of 633 nm are reported in Figure 4.

Here, a strong dependence of the Raman intensity at a distance from the tip is visible. In particular, the graph presents a bell-shaped behavior, with a maximum value obtained when the focus is adjusted at the tip. Otherwise, for focal planes above or below the antenna, the Raman intensity shows a clear decrease. This result demonstrates that the majority of the Raman signal comes from the tip of the antenna and this can be attributed to the presence of the hotspot reported in Figure 3. In order to quantify the enhancement factor, we compared the Raman signal from the tip of an antenna with the signal coming from the surrounding silver substrate.

**Figure 5:**
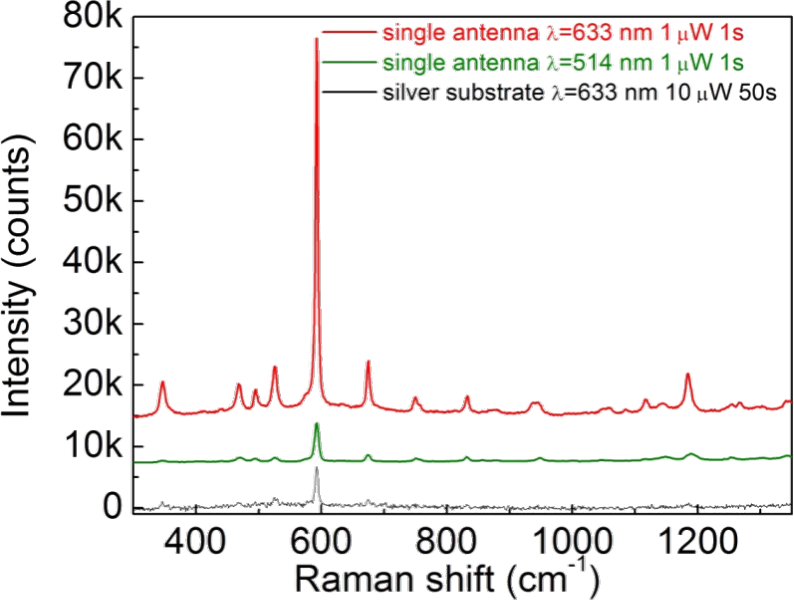
Raman scattering measurements of cresyl violet dye carried out on a rough silver substrate (black line) and single plasmonic nanoantenna (1.4 µm height and 80 nm radius) excited at a wavelength of λ = 633 nm (red line) and λ = 514 nm (green line).

Figure 5 reports the Raman spectrum of the probe molecule absorbed on a single antenna at excitation wavelengths of λ = 514 and λ = 633 nm and the Raman spectrum obtained on the silver substrate close to the antenna (thickness 30 nm, local roughness 4 nm). The laser power and acquisition time were 1 µW and 1 s, respectively, for the spectra acquired on the antenna, and 10 µW and 50 s for that on substrate. The acquisition time and laser power are the same for the spectra acquired at λ = 514 nm and λ = 633 nm. The spectra intensities were normalized with respect to the most intense Raman peak (593 cm^−1^).

Comparing the spectrum obtained on the antenna at λ = 633 nm (red line in Figure 5) with that on the substrate (black line in Figure 5), it is evident that the magnification of the Raman signals attributed to the antennas is much stronger than that of the silver film, thus confirming the effective capability of the hotspot at the antenna tip to act as field enhancer.

By taking into account the difference in laser power, integration time, signal counts, and number of scattering centers (hotspot area ≈10^3^ nm^2^, laser beam spot ≈2 × 10^6^ nm^2^) the estimated field enhancement factor of 60 was obtained. We remark that this value does not represent the absolute enhancement, but that one obtained in addition to that achievable on a rough silver surface. Since the latter is usually approximately 2–3 times the incident field or more, this implies that our antenna provides an experimental electromagnetic enhancement of about 120–180 in amplitude and is comparable to the result obtained by FEM calculations.

The data reported in Figure 5 allow quantification of the enhancement for a broadband wavelength range. Indeed, by comparing the spectral signals of the antennas, it is possible to observe that the dye excited at λ = 514 nm exhibits intensities 9.4 times lower than that measured at λ = 633 nm. Since the Raman intensity depends on the 4^th^ power of the local electric field amplitude, this means that at λ = 514 nm a field amplitude enhancement of 1.75 times lower than the one provided at λ = 633 nm relates to an absolute pure electromagnetic enhancement of about 70–100 times the amplitude of incident field. If we consider other Raman peaks, this estimation gives even higher values (>100).

This behavior is in complete agreement with the results obtained by calculations as reported in Figure 3, exhibiting a broadband absorption and efficient enhancement of the electric field over a wide spectrum. According to FEM simulations, the value of the electric field at λ = 633 nm was 200 times higher with respect to the incident field, while at λ = 514 nm, this value decreased to a value of 100. This is 2 times lower than the that obtained at a resonant wavelength and comparable to the experimental value.

Furthermore, Raman experiments have demonstrated that even though the antennas were optimized to work at 633 nm, they also provide high amplification of the electromagnetic field at different wavelengths, and the resulting enhancement is still higher than that derived from conventional broad-range plasmonic enhancers, such as silver nanoparticles [28]. Moreover, it has been verified that a fine tuning of the optical properties of antennas can be achieved by controlling their geometrical parameters such as height and diameter, in accordance with calculations derived from the FEM method.

## Experimental

Hollow three-dimensional electric field enhancing structures were obtained through an innovative fabrication method based on secondary electron lithography, generated by ion beam milling. The detailed process has been discussed elsewhere in depth [23]. Briefly, a layer of optical resist (Shipley S1813) is deposited by spin coating on the top of a silicon nitride membrane. The structure of the antenna is defined from the backside of the membrane by focused ion beam milling (FEI, NanoLab 600 dual beam system) using a gallium ion source with a current of 40 pA at a dose of 5 pC/μm^2^. The interaction between gallium ions and the photoresist produces high secondary electrons doses, which cause the inversion of a thin layer of resist around the milled hole. When the sample is immersed in acetone, unexposed resist is dissolved while the exposed polymer is insoluble and remains attached to the membrane, representing an exact replica of the milled structure.

Finite element method simulations were performed using the software COMSOL Multiphysics.

Raman spectra were recorded with a Renishaw InVia spectrometer using 633 nm and 514 nm laser sources. The antenna excitation and signal acquisition were carried out through a 100× objective at normal incidence in reflection configuration.

## Conclusion

In this work we demonstrated that engineered hollow plasmonic nanostructures can provide efficient electromagnetic field enhancement over a broadband spectrum. This could be a very attractive property for the design of highly versatile biosensors suitable for SERS spectroscopy on biological systems which use the entire visible wavelength range. The broadband enhancement feature is an addition to the already known broadband absorption of such systems [23].

FEM calculations were used to find suitable structure geometries by optimizing the value of electric field enhancement at 633 nm. It was found that a roughened silver nanoantenna presents an electric field enhancement value 200 times higher with respect to the incident field with respect to the optimal excitation wavelength. This value is far higher than values obtained for common plasmonic enhancers based on planar technologies.

In addition, hollow nanoantennas exhibit enhancement over a wide range of wavelengths – a feature not common in plasmonic systems. The simulation also revealed the presence of an electromagnetic field hotspot at the tip of the antenna confirmed by collecting the Raman spectra at different focusing distances along the main axis. A comparison of the Raman spectra obtained on antennas demonstrated an enhancement of the incident field of around 180 times. This was in accordance with the value calculated by FEM for the case of antennas excited on resonance and almost 2 times lower for out-of-resonance excitation. No consistent field enhancement was observed on the silver substrate close to antenna. These results are certainly encouraging and suggest the possibility of further integration of the nanocylinders in devices requiring multifunctional plasmonic properties over a broad wavelength range for the deep investigation of the complete cell environment.
